# Revolutionizing Seborrheic Keratosis Treatment: A Case Report of Successful Topical Treatment of Basal Cell Papilloma Using Siddha Medicine

**DOI:** 10.7759/cureus.80800

**Published:** 2025-03-18

**Authors:** Saravanasingh Karan Chand Mohan Singh, Ramamurthy Murugan, V Indumathy, Murugesan Sannasi

**Affiliations:** 1 Department of Maruthuvam, National Institute of Siddha, Chennai, IND; 2 Department of Noi Naadal, National Institute of Siddha, Chennai, IND; 3 Department of Siddha General Medicine, Sri Sairam Siddha Medical College and Research Centre, Chennai, IND; 4 Department of Siddha Toxicology, National Institute of Siddha, Chennai, IND

**Keywords:** basal cell papilloma, benign epidermal skin tumor, pachaieruvai, seborrheic keratosis, siddha medicine

## Abstract

Seborrheic keratosis (SK), also known as seborrheic warts or basal cell papilloma, is a common benign epidermal tumor typically seen in middle-aged and older adults. SK is one of the most frequently encountered skin lesions by primary care physicians and dermatologists in outpatient settings. Traditionally, cryosurgery and electrocautery have been the primary treatment options for SK. While both approaches are effective, these methods can result in unwanted side effects that may affect the cosmetic appearance of patients and can be quite costly, deterring some individuals from seeking treatment. Siddha external medicine provides a viable, cost-effective, and well-tolerated alternative. A 73-year-old female patient presented with black papules featuring fissures and ridges on a verrucous surface. The lesions, with a waxy "stuck-on" appearance, were located on the prominence of the concha of her left ear, measuring 1.5 cm in length and 6 mm in thickness. Though asymptomatic, the lesions raised significant cosmetic concerns. The diagnosis was confirmed through a thorough clinical evaluation. The Siddha formulation *Pachaieruvai* was applied externally for seven consecutive days. After one week of treatment, a significant reduction in both lesion thickness and pigmentation was observed. At the follow-up appointment, one week after the last treatment, the SK lesions were completely eradicated. No adverse symptoms or recurrence were noted. This research highlights the effectiveness of the Siddha approach to managing SK. Based on the findings, Siddha medicine proves to be an effective treatment for SK.

## Introduction

Seborrheic keratosis (SK) is a common benign skin condition characterized by immature epidermal keratinocytes. It is also referred to in the literature as a senile wart, seborrheic wart, verruca senilis, verruca seborrhoica, basal cell acanthoma, basal cell papilloma, or benign keratoacanthoma [1]. SK is a prevalent skin condition, affecting a large segment of the population. Studies show that up to 83% of individuals may develop at least one lesion by the age of 70 [2]. In Australia, the prevalence increases with age, reaching 100% in individuals older than 50 years [3]. In Korea, the prevalence among men aged 40-70 years is 88.1%, with a significant rise in both prevalence and lesion count as age advances [4]. Sex differences in prevalence have been observed, with some studies suggesting that women may have a higher rate of diagnosis due to greater awareness and concern for cosmetic appearance. However, other studies indicate no significant gender disparity [5]. Anatomically, SK lesions are most commonly found on sun-exposed areas such as the face, neck, and hands, while they are rarely seen on sun-protected regions like the palms and soles. This distribution highlights the role of ultraviolet (UV) radiation in the condition's development [1,4].

The exact etiology of SK is not well-established, though previous research has identified potential risk factors such as exposure to human papillomavirus (HPV) and UV light [6]. SK presents in various forms, ranging from lightly pigmented, superficial patches to brown or black, scaly papules or plaques with a characteristic "stuck-on" appearance. While treatment is not often necessary, many patients seek removal due to irritation, itching, or cosmetic reasons. SK can be effectively treated through several methods. Cryotherapy, which uses liquid nitrogen to freeze and eliminate lesions, demonstrates 100% efficacy but may cause side effects like hypopigmentation, particularly in individuals with darker skin [7]. Laser therapies, including erbium-doped yttrium aluminum garnet and CO_2_ lasers, offer high patient satisfaction and significant improvement, though temporary redness may occur [8]. For a less invasive option, topical treatments like hydrogen peroxide (especially at higher concentrations), calcipotriol, and tazarotene have proven effective. Calcipotriol, in particular, can achieve complete regression over several months [9,10]. Traditional electrodesiccation and curettage (ED&C) remains effective, though it may leave scars [11]. Plasma exeresis, a newer technique that uses high-frequency electrical energy, offers better clearance rates and fewer side effects compared to cryotherapy [12].

Siddha medicine adopts a holistic approach to treating dermatological conditions like SK, incorporating topical applications of kaaram, suttigai, seelai, or surgical removal. According to Siddha literature, kaaram involves the use of caustic chemicals for external growth removal. Derived from the ash of medicinal herbs, metalloids, and other natural sources, kaaram is commonly used by Siddha practitioners in the form of powder, paste, or aqueous solutions for localized treatment. This case report presents a pioneering instance in dermatology, where kaaram-Pachaieruvai was used to treat SK in a single patient.

This case report represents a significant milestone in dermatology, demonstrating kaaram as an effective treatment for SK, targeting conditions such as viral warts, moles, and cutaneous horns. The caustic nature of kaaram promotes the removal of unwanted tissues through localized sterile inflammation, acting as a sclerosing agent. As hyperpigmented papules undergo the sclerosis process, they gradually detach. Kaaram facilitates targeted and localized treatment, making it particularly effective.

## Case presentation

Background

A 73-year-old female patient presented with black papules characterized by fissures and ridges on a verrucous surface. These lesions had a waxy, "stuck-on" appearance and were located on the prominence of the concha of her left ear. Although asymptomatic, the lesions raised significant cosmetic concerns for the patient. Notably, her medical and family histories were unremarkable, and she had not received any prior treatments for this condition.

Clinical examination

Upon examination, the lesions were identified as well-demarcated, hyperkeratotic, warty, and pigmented, consistent with SK. The lesions measured 1.5 cm in length and 6 mm in thickness. The patient reported a history of prolonged sun exposure but denied any family history of similar lesions. The diagnosis was confirmed through a comprehensive clinical evaluation.

Siddha treatment

**Figure 1:**
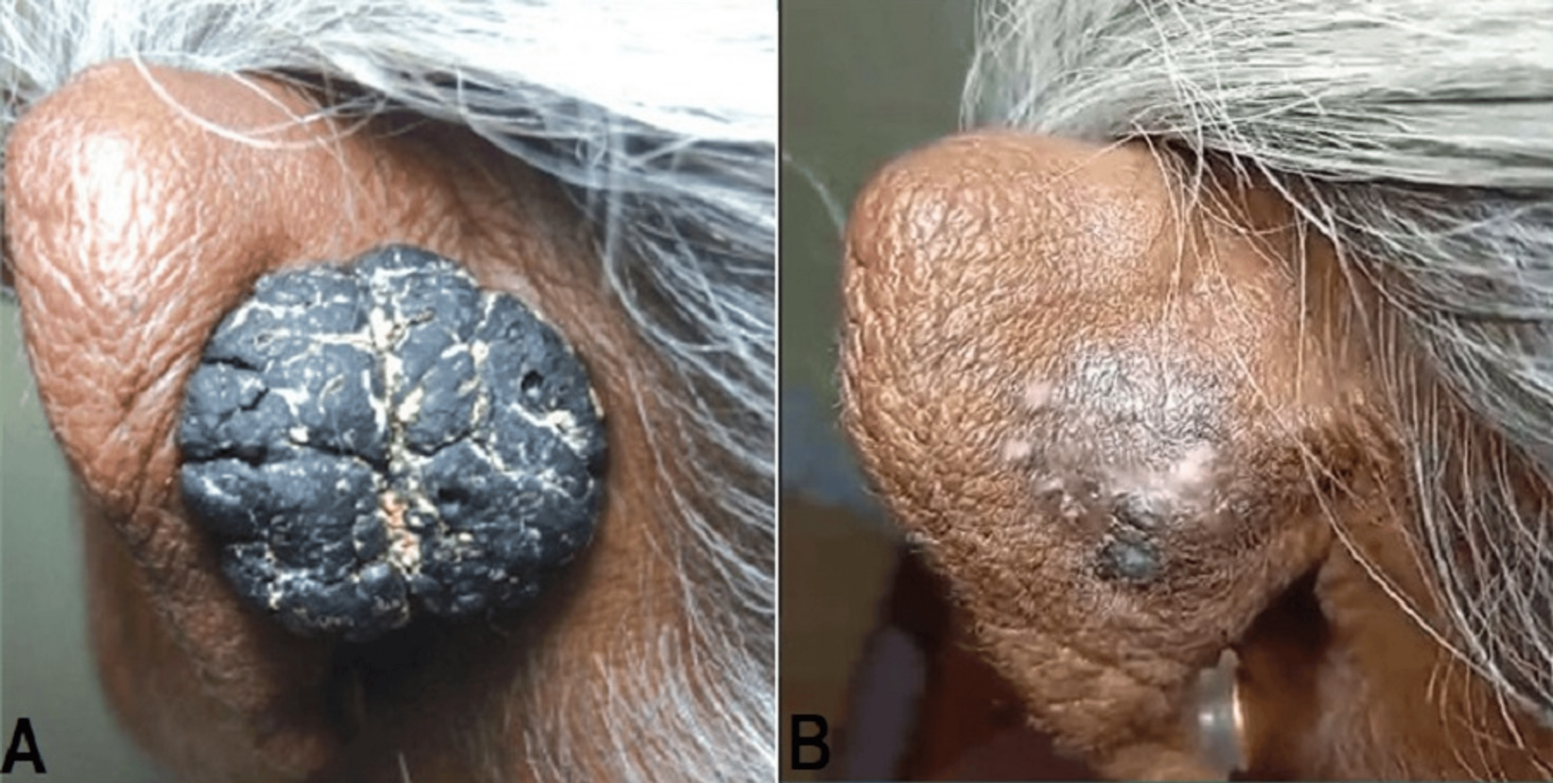
Seborrheic keratosis (A) before treatment and (B) 15 days after treatment

Treatment began after obtaining written consent from the patient. Pachaieruvai was applied externally for seven consecutive days, with adherence to the treatment protocol and continuous monitoring of the patient's response. A remarkable reduction in both lesion thickness and pigmentation was observed after just one week of treatment. At the follow-up appointment, one week after the final treatment, the SK lesions were completely eradicated. Additionally, no adverse symptoms or recurrences were reported, indicating the treatment's effectiveness and safety. The progress of the skin lesions on the ear before treatment and 15 days after treatment is shown in Figure 1.

## Discussion

This study provides an initial examination of the management of SK using Pachaieruvai, a traditional Siddha medicine, as outlined in the existing literature. Pachaieruvai contains vellaipadanam (arsenic trioxide), aridharam (arsenic trisulfide), thurusu (copper sulfate), karchunnam (calcium carbonate), and kungiliyam (resin of *Shorea robusta*) [13]. The use of Pachaieruvai offers several advantages, including cost-effectiveness, time efficiency, minimal dosage, and precise localized effects compared to other external treatments.

Research has shown that vellaipaadanam (arsenic trioxide), a key component of Pachaieruvai, inhibits the proliferation of human keratinocytes (HaCaT cells) by inducing apoptosis, as evidenced by DNA fragmentation, nuclear condensation, and activation of caspase-3 [14,15]. Aridharam (arsenic trisulfide) has cytotoxic properties that inhibit the development and growth of solid tumors [16]. Thurusu (copper sulfate) has cytotoxic effects that hinder tumor proliferation and possesses antiviral activity [17,18]. Karchunnam (calcium carbonate) exhibits cytotoxic properties, while kungiliyam (resin of *S. robusta*) has demonstrated cytotoxic effects that reduce cell proliferation [19,20].

Mode of action

The activation of the transforming growth factor beta 1 (TGF-β1) and extracellular signal-regulated kinase (ERK) pathways, along with the upregulation of BCL2-associated X protein (Bax), caspase-3, and caspase-9, suggests that the components of Pachaieruvai may suppress cellular proliferation by inducing apoptosis, as mentioned above.

Case benefits

The hyperkeratotic lesions on the ear were successfully treated, with complete eradication of the lesions. During the follow-up, no adverse symptoms or recurrences were reported, highlighting the treatment's effectiveness and safety.

Limitations of the study

Case studies focus primarily on individual patients, which presents challenges in extrapolating findings to larger populations. The presentation and severity of SK can vary significantly among individuals, making it inappropriate to generalize conclusions from a single case to all patients with the condition. While case studies can identify associations between causes and outcomes, they do not establish cause-and-effect relationships. The lack of controlled trials or extensive observational research makes it difficult to ascertain whether specific factors directly influence the development or progression of SK. Given the limited scope of case studies, they may not fully capture the range of SK manifestations or treatment responses.

Further research is needed to understand the variability of the condition comprehensively and to evaluate the efficacy of different therapies. There is a tendency to publish case studies highlighting uncommon or dramatic findings, which may introduce a bias toward extreme or atypical situations in the literature. This could create a misleading perception regarding the frequency and typical progression of SK. Nevertheless, case studies can provide valuable insights into therapy, inspire future research ideas, and illustrate the complexities of managing SK in real-life scenarios. Caution should be exercised when interpreting these findings, and they should be complemented with data from other study designs to gain a comprehensive understanding.

## Conclusions

The hyperkeratotic lesions on the ear were treated successfully, resulting in complete eradication. The external application of Pachaieruvai, a traditional Siddha medicine, proves effective in treating SK. Pachaieruvai is cost-effective and well-tolerated, emphasizing the potential of Siddha medicine in managing SK. No adverse effects were noted with the prescribed treatment. However, further studies are needed to confirm these findings and explore the potential role of Pachaieruvai in SK management. Larger studies are essential to establish Siddha medication as an effective approach for managing SK.
